# Preoperative clinical factors and visual outcomes following orbital decompression with dysthyroid optic neuropathy

**DOI:** 10.1186/s12886-020-1314-8

**Published:** 2020-01-17

**Authors:** Mizuki Tagami, Shigeru Honda, Atsushi Azumi

**Affiliations:** 1 Department of Ophthalmology, Visual Sciences Graduate School of Medicine, Osaka City University, 1-5-7 Asahimachi, Abeno-ku, Osaka-shi, 545-8586 Japan; 2 Ophthalmology Department and Eye Center, Kobe Kaisei Hospital, Kobe, Hyogo Japan

**Keywords:** Graves’ orbitopathy, Orbital decompression, Dysthyroid optic neuropathy, Visual outcome, Japanese

## Abstract

**Background:**

To investigate preoperative clinical factors and visual outcomes of Japanese patients with dysthyroid optic neuropathy (DON) after urgent orbital decompression.

**Methods:**

This retrospective, observational case series study investigated 44 patients who exhibited several preoperative clinical factors that might be associated with the need for urgent orbital decompression due to DON. Additionally, the visual acuity of DON patients was compared between the patients preoperatively and at 1 and 6 months postoperatively.

**Results:**

All 44 patients received steroid and with or without radiation therapy, with 27 patients able to avoid undergoing urgent surgery. However, the remaining 17 patients required urgent orbital decompression following a lack of response to the therapy. None of the patients who initially avoided surgery required additional surgery for DON. Factors significantly associated with the need for urgent orbital decompression surgery included: female gender, older age, long disease duration, unilateral significant DON, history of resistance to pulsed steroid therapy, unstable thyroid function, high TRAb (Thyrotrophin receptor antibody)value, poor visual acuity, presence of central diplopia, and presence of corneal problems (*P* < 0.05 each). The results also showed that postoperative visual outcomes of surgery for DON were acceptable.

**Conclusion:**

This study revealed several preoperative clinical factors for DON that appear to be associated with the need for urgent orbital decompression surgery in Japanese patients.

## Background

Graves’ orbitopathy (GO) is a potentially sight-threatening ocular disease, with the associated dysthyroid optic neuropathy (DON) particularly leading to blindness. Optic neuropathy may also cause a loss of vision in patients with GO [1, 2]. DON is an uncommon manifestation of GO that affects approximately 5% of patients with GO [3]. The 2016 European Thyroid Association/European Group on Graves’ Orbitopathy Guidelines for the Management of Graves’ Orbitopathy (EUGOGO) recommended treatment with high-dose intravenous corticosteroid for 2 weeks. In the event of a poor treatment response, prompt orbital decompression is required [4].

The present case series investigated methods used to avoid urgent surgery by comparing preoperative clinical factors and visual outcomes in an urgent surgery group of patients with compressive optic neuropathy. This study also investigated factors that could potentially be used to determine the need for urgent surgery, as well as the efficacy of orbital decompression for DON.

## Methods

Prior to the start of this study, approval was obtained from the Institutional Review Board at Kobe Kaisei Hospital in Japan. Written informed consent for the storage of patient information in the hospital database and use in this research was provided by all patients enrolled in the study. The study was performed according to the tenets of the Declaration of Helsinki.

Our treatment schedule for DON consisted of a cumulative dose of 9.0 g of methylprednisolone, which was divided into 3 weekly infusions, and combined with 20 Gy of radiation therapy in 10 fractions. After this treatment, methylprednisolone was gradually reduced from a dose of 0.5 mg/kg/month over a period of 6 months. Patients in this study who underwent urgent orbital decompression were followed for at least 6 months postoperatively. Urgent orbital decompression for DON was defined as a surgery performed within 2 weeks of the initial visit to our hospital. We reviewed the results of the clinical ophthalmic examinations, which included best-corrected visual acuity (BCVA), intraocular pressure, exophthalmometric measurements performed using a Hertel exophthalmometer, a Hess screen test, a binocular single vision test, and magnetic resonance imaging (MRI) performed with and without gadolinium contrast. None of the patients examined had claustrophobia or any previous brain aneurysm clip implants. Serological data including TARb (Thyrotrophin receptor antibody) was also analyzed. DON was defined as follows: 1) a history of thyroid disease; 2) visual loss of < 0.1 logMAR, or central critical fusion frequency (CFF) < 30 Hz; and 3) presence of orbital apex crowding on a MRI scan. Exclusion criteria were as follows: 1) history of traumatic head injury; 2) history of brain tumor; 3) presence of high myopia > − 9.0 D; 4) history of glaucoma involving ocular hypertension; or 5) systemic neuropathy in the active phase. Inclusion criteria for urgent surgery at our institution were as follows: 1) visual acuity < 0.1 logMAR; 2) CFF ≤20 Hz; and 3) presence of any scotoma or visual field defect that has not improved after 2 weeks of steroid treatment.

Two surgeons (A.A., M.T.) performed the orbital decompression surgery in all patients under general anesthesia, as per a previously reported method [2]. Specifically, decompression of the deep lateral orbital wall was performed via an eyelid crease incision. In most patients, the greater wing of the sphenoid bone was removed along with the additional removal of the anterior compartment of the inferior orbital fissure. Medial orbital decompression was performed via a transcaruncular incision. After exposure of the medial wall immediately posterior to the posterior lacrimal crest, the wall was fractured, and anterior and posterior ethmoidectomies were performed. The ethmoidal vessels were used as the superior limit of the medial wall removal, while the inferior limit included the ethmoid-maxillary bony strut. Microscopy was used in all procedures.

The corrected decimal BCVAs were converted to the LogMAR scores for statistical analysis. Statistical analyses were performed using SPSS Statistics version 22 software (IBM Japan, Tokyo, Japan). Values of *P* < 0.05 were considered statistically significant.

## Results

This study included 44 consecutive Japanese GO patients with DON who were examined at the Ophthalmology Department of the Eye Center at Kobe Kaisei Hospital in Kobe, Hyogo, Japan between January 2011 and October 2016. Among them, ten patients had received steroid therapy or radiation therapy, or both for GO in the past. Of the 44 patients (70 eyes) with DON who were examined in the study, 27 patients (46 eyes) were able to avoid urgent surgery due to the use of steroid and radiation therapy. The remaining 17 cases (24 eyes) were enrolled in the study as members of the urgent surgery group. None of the patients who initially avoided urgent decompression later required any additional surgery for DON.

Mean duration of postoperative follow-up for the 17 patients (24 eyes) who underwent urgent orbital decompression was 40.9 ± 21.2 months (range, 6–72 months). Table 1 presents the demographics and clinical features of patients included in the study. There were no records of any thyroid surgery before DON treatment. There were four patients after RAI, two patients for each group. Factors that were significantly associated (*P* < 0.05; Table 1) with the need for urgent orbital decompression surgery included: female gender, older age, long disease duration, unilateral significant DON, history of resistance to treatment with pulsed steroid therapy, unstable thyroid function, TRAb Value, poor visual acuity, presence of central diplopia, and the presence of corneal problems. There were no other preoperative factors that were significantly associated with the need for urgent surgery (*P* > 0.05). The surgical procedures performed included 10 cases of unilateral medial decompression, 5 cases of bilateral medial decompression, and 2 cases of bilateral balanced decompression (deep lateral and medial decompression).

**Table 1:** Preoperative characteristics of 44 patients with Graves’ orbitopathy and dysthyroid optic neuropathy

Parameter	Total	Without surgery	Urgent surgery	*P*
Number of patients (male/female)	44 (15/29)	27 (9/18)	17 (6/11)	0.014*
Age, years	53.4 ± 8.9	47.1 ± 6.7	59.7 ± 13.2	< 0.01*
Disease duration, months	4.8 ± 5.0	3.1 ± 6.6	6.5 ± 4.1	< 0.01*
Laterality of GO				
Bilateral	26	19	7	< 0.0
Unilateral	18	8	10	1*
Number of treatments with pulsed steroid therapy	2.6 ± 2.1	3.2 ± 1.1	2.1 ± 1.3	< 0.01*
Orbital radiation therapy				
+	24	16	8	< 0.01
-	20	11	9	*
Thyroid function				
Stable	32	20	12	0.013
Unstable	12	7	5	*
TRAb IU/l	16.9 ± 25.7 20	11.4 ± 15.4 11	43.9 ± 44.2 9	0.04*
NA				
Visual acuity, logMAR conversion	0.15 ± 0.33			
Better	0.71 ± 0.71	0.12 ± 0.46	0.19 ± 0.27	< 0.01
Worse		0.66 ± 0.55	0.84 ± 0.79	*
Diplopia, central 30 degrees				
+	17	11	6	< 0.01
-	22	16	9	*
NA	2		2	
Asymmetry of proptosis, mm	1.4 ± 1.3	1.3 ± 1.5	1.4 ± 0.8	0.26
Exophalmos degree	17.8 ± 1.8	18.1 ± 1.9	17.2 ± 1.2	0.06
Secondary high IOP				
+	8	4	4	0.15
-	23	11	12	
NA	13	10	3	
Corneal problems				
+	16	8	8	
-	15	9	6	0.22
NA	13	10	3	
Psychological history				
+	9	6	3	0.39
-	35	21	14	
Smoking history				
+	10	6	4	
-	27	14	13	0.29
NA	7	7	0	

A t-test was used to analyze the data on smoking, while diplopia data were analyzed with the χ^2^ test. These data are expressed as mean ± standard deviation

TRAb: Thyrotrophin receptor antibody

**Fig. 1:**
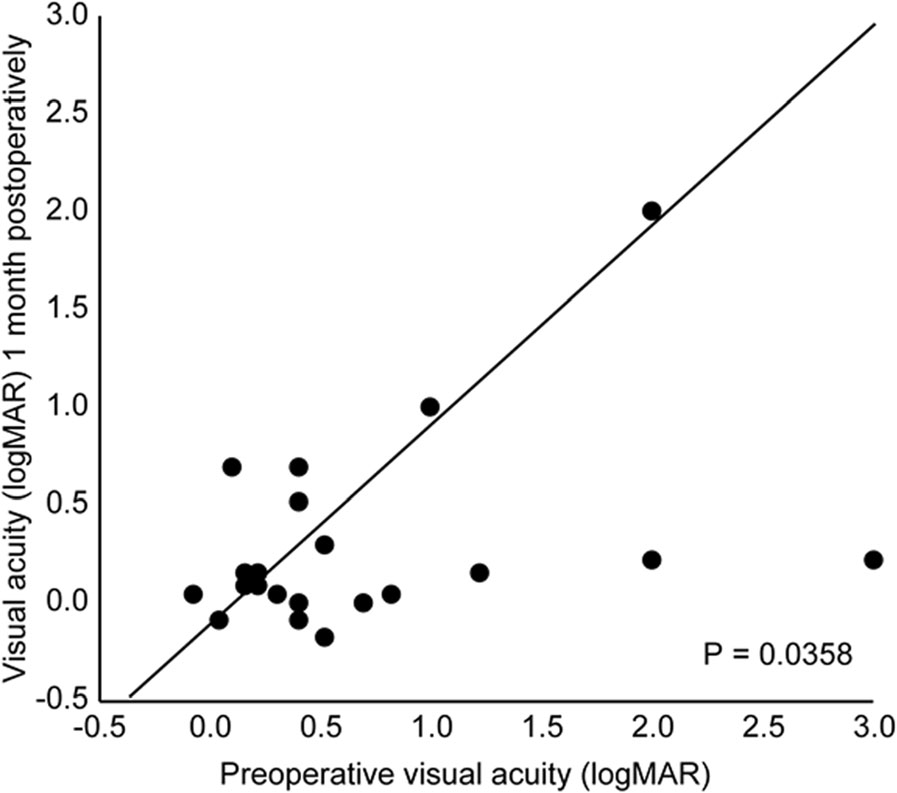
Pre- and postoperative (1 month after orbital decompression) visual acuity. Eyes in which visual acuity improved following orbital decompression are located below the diagonal line. *P*-value, repeated-measures analysis of variance, *n* = 24

**Fig. 2:**
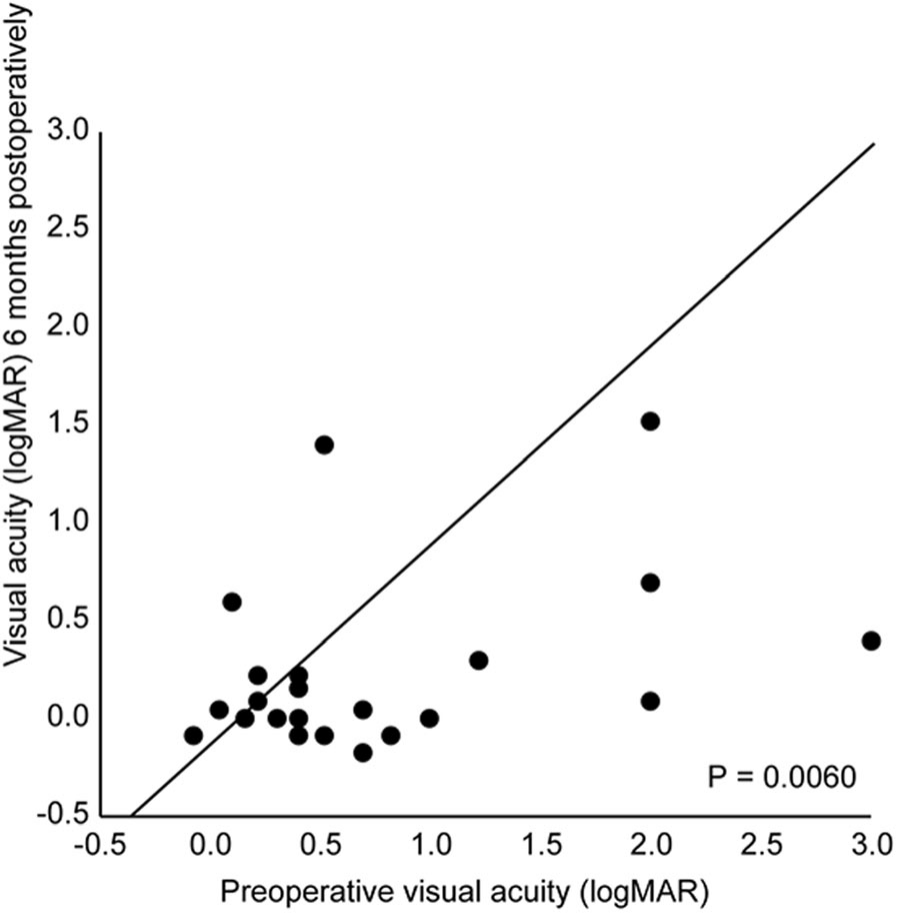
Pre- and postoperative (6 months after orbital decompression) visual acuity. Eyes in which visual acuity improved following orbital decompression are located below the diagonal line. *P*-value, repeated-measures analysis of variance, *n* = 24

In urgent surgery cases, the mean BCVA immediately prior to surgery was 0.73 logMAR (standard deviation [SD]: 0.77). Following surgery, significant changes in the BCVA were observed at 1 month postoperatively (*n* = 24; 0.33 logMAR; SD 0.58; *P* < 0.05) (Fig. 1) and at 6 months postoperatively (n = 24; 0.21 logMAR; SD 0.43; *P* < 0.010) (Fig. 2). Out of the 17 patients, 7 (42%) did not achieve a visual acuity of 0.1 logMAR by 6 months following the urgent decompression. Table 2 summarizes the characteristics and reasons of visual outcomes of these patients.

**Table 2:** Clinical characteristics of patients who did not achieve a visual acuity of 0.1 logMAR by 6 months after urgent decompression

No.	Sex	Age range	Duration before surgery (months)	Type of decompression	Number of pulsed steroid treatments	RT	Smoking history	Comment
	1	60–65	13	Bilateral medial decompression	1000 mg 3 days ×2 treatments	Done	–	1. Psychological factor (depression) 2. Central scotoma
2	1	75–80	12	Unilateral medical decompression	None	Done	–	1. No steroid pulses due to gastric cancer 2. Cataracts
3	1	50–55	3	Bilateral balanced decompression	None	None	+	1. Rejection of steroid pulse for work 2. Postoperative visual loss
4	2	80–85	13	Unilateral medical decompression	1000 mg 3 days ×4 treatments	None	–	Diabetic macular edema
5	2	55–60	6	Unilateral medical decompression	1000 mg 3 days ×3 treatments	None	–	Cataracts
6	2	70–75	7	Bilateral medial decompression	1000 mg 3 days × 3 treatments	Done	–	Diabetic retinopathy Cataracts
7	2	70–75	9	Unilateral medical decompression	1000 mg 3 days ×3 treatments	Done	+	Unknown origin. Improved gradually to 0.1 logMAR visual acuity.

*RT* radiation therapy

## Discussion

Orbital decompression is a common and major treatment option for compression optic neuropathy that is resistant to immunosuppression and radiation therapy [4–7]. However, general anesthesia is typically required and surgical complications are not uncommon. Jacobs et al. reported that causes of vision loss following orbital surgery included retrobulbar hemorrhage, a malpositioned implant, optic nerve ischemia, or direct optic nerve insult, with the overall risk of severe vision loss found to be 0.84% [8]. Although this previous report discussed the complications of orbital surgery in general, the approach they used for orbital decompression surgery was similar to our own methodology, and thus served as a reference for our current work. A previous report that examined follow-up surveys for 215 patients with Graves’ optic neuropathy who underwent surgical treatment at the Mayo Clinic between 1969 and 1989 remains, to the best of our knowledge, the largest reported series of patients with Graves’ optic neuropathy [9]. The majority of patients with Graves’ optic neuropathy were women, with a reported 2.4:1 ratio of women to men. Patients with optic neuropathy had a median age at onset of ocular symptoms that was 11 years older than that of patients without optic neuropathy. Some reports have also examined medical treatments including the use of steroids for severe DON. The most common schedule for intravenous glucocorticoid (GC) therapy, which is based on a randomized clinical trial, is a cumulative dose of 4.5 g of methylprednisolone divided into 12 weekly infusions (6 weekly infusions of 0.5 g, followed by 6 weekly infusions of 0.25 g) [10].

In a large, multicenter EUGOGO randomized clinical trial that included 159 patients with moderate-to-severe and active GO, three different cumulative doses of methylprednisolone were used (7.47, 4.98 and 2.25 g) over the same time period [11]. Although the clinical activity score decreased significantly for all doses, overall ophthalmic improvement was significantly more common in the group with the highest dose (52% vs. 35 and 28% when using the other two doses, respectively). Even so, the highest dose was associated with a slightly greater frequency of adverse events; with a high single (> 0.5 g) and/or cumulative dose (> 8 g) of intravenous GCs associated with a doubling of the rate of adverse events i.e. viral pneumonia (56% vs. 28%, *P* < 0.001, and 52% vs. 33%, *P* = 0.034, respectively) [12]. However, high daily doses (0.5–1.0 g) administered several times per week for 2 consecutive weeks are generally necessary in patients with sight-threatening GO [1, 4]. In cases of DON, we believe that prompt surgical consultation is necessary if medical treatment appears ineffective. Therefore, although medical intervention is often required, it is necessary to start with a cumulative dose of 9.0 g of methylprednisolone, which is then divided into 3 weekly infusions.

The present study compared the preoperative clinical features of DON with optic nerve compression between patients who underwent urgent surgery and those who did not. We found that female gender, older age, long disease duration, unilateral significant DON, unstable thyroid function, high TRAb value, and poor visual acuity were factors significantly associated with the need for surgical intervention. Jack Rootman stated in his book on orbital surgery that although typical GO is four times more common in women than it is in men, severe cases are more commonly observed in men [13]. Although the reason for this was not stated, differences in rates of smoking, among other factors, might be involved. The observed ratio of men to women found in the present study was lower than that which has been previously reported.

Interestingly, some authors have found that smoking represents an important factor for the reactivation of GO [5]. Smoking is a well-known risk factor for GO, with a previous case-control study reporting an odds ratio of 7.7 for the association between smoking and the incidence of GO [14]. However, we did not find any significant association between a history of smoking and the need for surgical intervention in the present study.

Another feature of this study is that the higher the value of TRAb, the more likely necessary to urgent surgery. In a few reports, the relevance of TSAb (thyroid stimulating antibody) to DON has been described in previous case series [15]. This is a clinical bio-marker that is significant in clinical practice in GO.

Regarding visual acuity, a previous report examined the visual acuity of 383 eyes both preoperatively and during the early postoperative period (182 days). In this previous study, 69 eyes (18%) showed a visual acuity of 20/20 prior to decompression as compared to 125 eyes (33%) that had the same visual acuity during the early postoperative period [9].

In the present study, we found significant changes in the BCVA between the values seen preoperatively and those at both 1 and 6 months postoperatively. At 6 months postoperatively, 10 of the 17 patients (58%) achieved a visual acuity better than 0.1 logMAR. These positive outcomes may be related to improvements in the surgical methodology that have occurred since the initial previous reports described the original transantral orbital decompression procedure [9, 16]. In contrast, the present study used transcaruncular medial decompression and deep lateral orbital decompression. We hypothesized that we would find a reduction in the organ damage and pressure in our current work as compared to that which has been reported in previous studies. Furthermore, no cases of severe vision loss after surgery were seen in any of the patients included in our study. This again may be due to the microsurgical methods used in the present study.

In terms of the visual prognosis for the 17 patients, 7 (42%) did not achieve a visual acuity of 0.1 logMAR by 6 months postoperatively. However, when the clinical courses of these patients were evaluated in detail, 3 of the patients were found to have cataracts, with visual acuities improving to 0.1 logMAR after cataract surgery, while 2 patients had diabetic maculopathy. In another patient who was found to have a visual acuity of worse than 0.1 logMAR immediately following surgery, visual acuity gradually increased after starting dry eye therapy. Thus, this patient may have had superior limbic keratoconjunctivitis, which has previously been reported in patients with Graves’ disease [17]. Among all of the patients examined in this study, only 1 patient had a visual acuity of worse than 0.1 logMAR, which was secondary to compressive optic neuropathy. Consequently, 16 of the 17 patients (94%) achieved visual acuities of 0.1 logMAR by the time of their final visits. This result is in accordance with the findings of a previous report [18].

Some of the limitations that need to be considered when interpreting the results of the present study include the small sample size, the absence of a control group, and the single-center nature of this case series. Additionally, several confounding factors were present in this study, including the fact that there was a correlation between the preoperative low vision and the urgent operation group. Another possible flaw is that normal-tension glaucoma (NTG) occurs in many Japanese patients. As not all of the visual field abnormalities could be completely distinguished from NTG, this remains a limitation of the present study [19].

We acknowledge these potential issues as well as the need for future worldwide studies with objective data that compare this surgery with treatments that use corticosteroids and radiation therapy.

## Conclusion

This study revealed several preoperative clinical factors for DON that appear to be associated with the need for urgent orbital decompression surgery in Japanese patients. In addition, 16 of the 17 patients (94%) achieved visual acuities of 0.1 logMAR by the time of their final visits in urgent surgery group.

## Data Availability

All data included in this study are available from the corresponding author on reasonable request.
